# Comparative evaluation of AI language models in educating patients on women’s sexual health

**DOI:** 10.1177/17562872251407371

**Published:** 2026-01-02

**Authors:** Yash H. Kadakia, Muhammed A. M. Hammad, Elia Abou Chawareb, Faysal A. Yafi, Olivia H. Chang, Jessica M. Yih

**Affiliations:** Department of Urology, University of California, Irvine, CA, USA; Department of Urology, University of California, Irvine, CA, USA; Department of Urology, University of California, Irvine, CA, USA; Department of Urology, University of California, Irvine, CA, USA; Department of Urology, University of California, Irvine, CA, USA; UC Irvine Manchester Pavilion, 200 S Manchester Ave, Orange, Irvine, CA 92868, USA

**Keywords:** artificial intelligence, large language models, patient education

## Abstract

**Background:**

Artificial intelligence (AI) is increasingly used in patient education, especially with the rise in popularity of large language models (LLMs) such as ChatGPT, Microsoft Copilot, and DeepSeek, offering quick, accessible answers to health-related queries. Yet, in female sexual health, a field historically under-researched and stigmatized, AI’s role in patient-facing education has yet to be thoroughly explored.

**Objectives:**

To evaluate the accuracy and relevance of responses from ChatGPT, Copilot, and DeepSeek to common female sexual health questions, comparing them to the Prosayla website and to each other.

**Design and methods:**

Twelve questions were developed based on content from the Prosayla website, covering topics ranging from menopause to sexual dysfunction. Responses were collected from the three LLMs and Prosayla. Two female sexual medicine experts independently rated each response for accuracy and relevance utilizing a six-point Likert scale (0–5) with a double-blind design being used to minimize bias. One-way ANOVA and Bonferroni post hoc analyses were used to assess statistical significance (*p* < 0.05).

**Results:**

No significant differences in accuracy scores were observed across the four sources for Physician A (*p* = 0.558) or Physician B (*p* = 0.052), although ChatGPT was rated significantly more accurate than Prosayla in post hoc analysis by Physician B (*p* = 0.044). Relevance scores differed by rater: Physician A found no differences across sources (*p* = 0.771), while Physician B rated all three AI models significantly higher in relevance than Prosayla (*p* < 0.001).

**Conclusion:**

AI models demonstrated comparable accuracy to Prosayla (a trusted patient education source), with the models being more relevant for one of the raters. These findings suggest that AI tools may complement traditional educational materials and support patient learning. However, expert oversight remains essential to ensure content quality and appropriateness. Future efforts should develop structured strategies and implementation frameworks to responsibly integrate AI into patient education, particularly in sensitive areas like women’s sexual health.

## Introduction

Artificial intelligence (AI) has become an increasingly popular tool for medical advice, especially since the release of the popular large language model (LLM) ChatGPT.^1^ LLMs such as ChatGPT are widely accessible and have straightforward, user-friendly interfaces, incentivizing consistent usage. AI is emerging as a powerful adjunct, aiding in diagnosis, education, and decision-making processing. Recent evidence highlights the growing role of AI in sexual health, noting its applications in sexually transmitted infection prevention, infertility treatment, and even sexual dysfunction diagnostics.^2^ This potential is further supported by findings from urological research on urolithiasis and prostate cancer, where ChatGPT has been shown to deliver information with accuracy comparable to traditional patient resources.^3^ In fact, urologists in most cases evaluated that favored AI responses for their clarity and usefulness, underscoring its promise in enhancing patient comprehension.

While the increasing popularity of AI has been explored in many urological and non-urological fields alike, there is still a need to see its impact specifically on female sexual health. Historically, female sexual health has received limited research attention compared to its male counterpart, with similarly many barriers being present for midlife women trying to access sexual health services.^4^ Furthermore, this is amplified by the social stigma around female sexuality, making some uncomfortable discussing their sexual health even with their healthcare professionals.^5^ These gaps underscore the need to assess whether AI-generated educational content can serve as a reliable, accessible resource for women seeking information on sensitive sexual health concerns; an aim that underpins the present study. To provide a meaningful benchmark, the study compared the responses generated by the LLMs to Prosayla, which is a traditional patient education website supported by the International Society for the Study of Women’s Sexual Health (ISSWSH). Prosayla reflects guideline-informed, expert-reviewed material that is commonly recommended to patients. This makes it a relevant and credible comparator for evaluating the accuracy and relevance of AI-generated information.

## Materials and methods

Twelve frequently asked questions (Table 1) regarding women’s sexual health were developed for evaluation by two female sexual health urologists. The questions focused on common patient concerns across topics, including menopause, sexual pain disorders, orgasmic disorders, desire disorders, and pelvic pain. The questions were developed after an analysis of the articles on the Prosayla website (patient education source supported by ISSWSH) and refined after feedback from the physicians based on what was seen in clinical practice. Since Prosalya reflects ISSWSH guideline-based content, it served as a clinically validated benchmark for evaluating the quality of AI-generated educational material. The questions were input into the web-interface versions of three prominent AI LLMs: ChatGPT, CoPilot, and DeepSeek. For ChatGPT, the free version was being utilized, which meant the responses were being generated with a combination of their GPT-3.5 and GPT-4 models. The free version of Microsoft Copilot was powered by both GPT-4 and their own model when responding. For DeepSeek, the free version was used as well, with their DeepSeek-V3 being utilized to generate responses. All AI model queries and Prosayla extractions were conducted between November 2024 and March 2025. Although the study period spanned several months due to question refinement, all final queries were conducted using the same freely available versions of ChatGPT (GPT-3.5/GPT-4), Microsoft Copilot (GPT-4 hybrid), and DeepSeek-V3 to ensure consistency in model versions. Each chatbot was instructed to keep its response limited to 2–3 sentences to ensure that the formatting of the responses was uniform across each of the sources. This was done by prompting each chatbot to “keep responses to 2-3 sentences.” The reasoning behind this was to ensure that all responses across the LLMs were standard and comparable. The responses from each of these chatbots were compared against the information provided on the Prosayla website, a trusted resource supported by ISSWSH, as well as to one another for consistency and variation. Responses from Prosayla were copied verbatim from relevant parts of certain articles that related to the questions proposed.

**Table 1.** Frequently asked questions regarding women’s sexual health are used for evaluation by physicians.

Questions
Question 1: How does menopause affect sexuality?
Question 2: What is hypoactive sexual desire disorder?
Question 3: What role does sleep play when it comes to my sexual health?
Question 4: What is Genitourinary Syndrome of Menopause?
Question 5: Why do I have pain when having sex?
Question 6: What can I do to have normal sex after cancer?
Question 7: What is causing my pelvic floor discomfort?
Question 8: Who do Clitoris Adhesions affect?
Question 9: What is the cause of lichen sclerosus?
Question 10: When do people with Vestibulodynia feel pain?
Question 11: Is there a cure for vulvovaginal lichen planus?
Question 12: What should I do if I think I have a vulvo-vaginal skin disorder?

The outputs from all four sources (ChatGPT, Copilot, DeepSeek, and Prosayla) were independently evaluated by the two sexual health specialists. Each response was rated utilizing a six-point Likert scale (0–5). A score of 0 indicated poor reliability or accuracy, while a score of 5 indicated excellent reliability or accuracy. Two domains were assessed: (1) the clinical accuracy of the information and (2) the overall relevance of the response to the question asked. A score of 5 in either domain reflected information that was factually correct, comprehensive, and either clinically sound or highly relevant to the patient’s question. By contrast, a score of 0 indicated that the response lacked accuracy or relevance entirely, with information that was incorrect, misleading, or not responsive to the question.

To ensure that bias was not a factor, the study was conducted in a double-blind fashion where the physicians were blinded to the identity of the source of each response and were also blinded to each other’s evaluations. This methodology ensured independent assessment and minimized the potential for external influence on the ratings.

A one-way analysis of variance (ANOVA) was conducted to assess whether there were statistically significant differences in the Likert scale ratings of accuracy and relevance among the four response sources: ChatGPT, Copilot, DeepSeek, and Prosayla. Bonferroni was used for post hoc analysis to evaluate specific group differences while controlling for multiple comparisons. This ensured that Type 1 error was minimized across pairwise evaluations.

## Results

A total of 12 questions were evaluated across four sources: ChatGPT, Copilot, DeepSeek, and Prosayla. The responses were independently rated for both accuracy and relevance by two female sexual health experts utilizing a 0–5 Likert scale.

### Accuracy

There were no statistically significant differences in accuracy ratings across the four sources (ChatGPT, Copilot, DeepSeek, and Prosayla) for either physician. The one-way ANOVA analyses for Physician A yielded *p* = 0.558 (Table 1) and for Physician B yielded *p* = 0.052 (Table 2). However, Bonferroni’s post hoc testing for Physician B revealed a statistical difference between ChatGPT and Prosayla, with ChatGPT achieving higher accuracy (*p* = 0.044). Outside of this, there were no other comparisons between the four responses’ sources that were statistically significant (Table 3).

**Table 2.** Physician A ANOVA accuracy (*p* = 0.558).

Source	Mean	Std. Dev	Significant compared to Prosayla
Prosayla	4.83	0.389	No
ChatGPT	5.00	0.000	No
Copilot	4.92	0.289	No
DeepSeek	4.92	0.83	No

ANOVA, analysis of variance.

**Table 3.** Physician B ANOVA accuracy (*p* = 0.052).

Source	Mean	Std. Dev	Significant compared to Prosayla
Prosayla	4.50	0.674	Yes
ChatGPT	5.00	0.000	No
Copilot	4.83	0.389	No
DeepSeek	4.83	0.459	No

ANOVA, analysis of variance.

### Relevance

**Figure 1.**
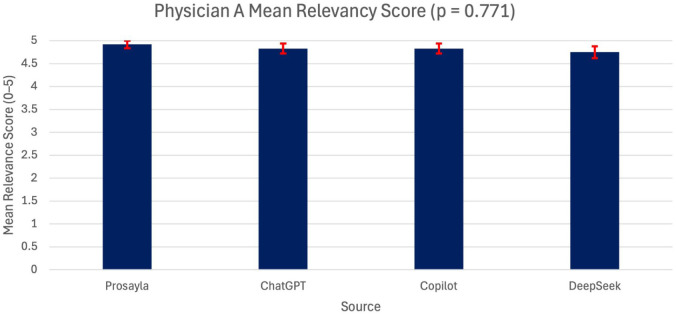
Mean relevance scores assigned by Physician A across all four sources (Prosayla, ChatGPT, Copilot, and DeepSeek). No statistically significant differences were found between sources (ANOVA, *p* = 0.771). Error bars represent the standard error of the mean, and *n* = 12 questions were scored. ANOVA, analysis of variance.

**Figure 2.**
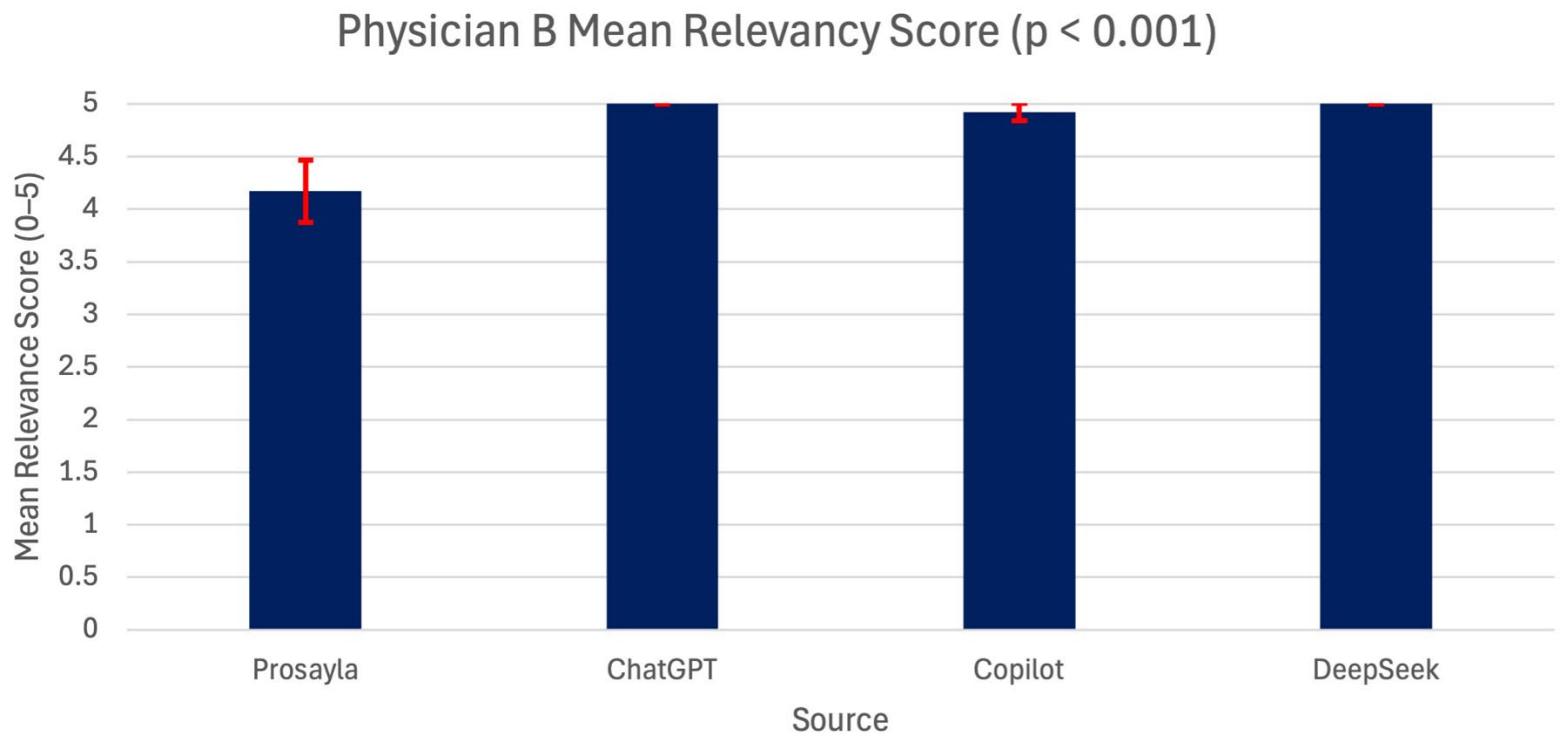
Mean relevance scores assigned by Physician B across all four sources (Prosayla, ChatGPT, Copilot, and DeepSeek). A statistically significant difference was observed between sources (ANOVA, *p* < 0.001), with Prosayla rated notably lower in relevance. Error bars represent the standard error of the mean, and *n* = 12 questions were scored. ANOVA, analysis of variance.

In contrast, a more distinct pattern emerged when examining relevance scores. For Physician A, there were no statistically significant differences across the sources (*p* = 0.771) (Figure 1). However, for Physician B, a significant difference was observed with *p* < 0.001 (Figure 2). The post hoc Bonferroni testing highlights that all three AI models—ChatGPT, Copilot, and DeepSeek—were rated significantly higher in relevance compared to Prosayla (*p* < 0.01 for each comparison).

## Discussion

As AI becomes prominent in many aspects of life, the question has arisen as to how prominent a role it should play in medicine.^6^ This question is especially relevant when it comes to female sexual health, an area that has been historically under-researched, where accurate and relevant patient education is crucial. Trusted patient education resources, such as Prosayla, have served as helpful guides. But since these guides are in the form of website articles, they can be limited in the scope of specific questions they can answer, as each patient’s needs are different.^7^ This could mean that there could be many articles that are not relevant to an individual patient. However, as demonstrated in this study, LLM models like ChatGPT, Copilot, and DeepSeek may offer comparable or even enhanced educational content in certain dimensions, particularly relevance. This may be because LLMs offer real-time customization, tailoring responses to the exact question being asked rather than providing broad, pre-written content. Their ability to generate concise answers in a conversational tone can also make the information feel more approachable and easier to understand. Furthermore, the brevity of AI responses may help users quickly find the specific information they are seeking without having to sift through lengthy articles.

Our findings suggest that the accuracy of AI-generated responses is comparable to that of traditional resources. Both Physician A and Physician B rated the accuracy of responses similarly across ChatGPT, Copilot, DeepSeek, and Prosayla. Even though Bonferroni’s post hoc analysis revealed ChatGPT to have statistically significantly higher accuracy ratings than Prosayla for Physician A, the overall pattern highlights that AI models can produce medically accurate information at a level similar to conventional sources. These results align with recent literature highlighting that LLMs can deliver medically accurate information to patients, underscoring their potential role in patient education.^8^

Regarding relevance, our findings were more nuanced. Physician B found significant differences favoring AI models over Prosayla, while Physician A did not observe any significant variation. Several factors could explain this discrepancy. One consideration is the difference in clinical backgrounds and areas of expertise between the two physicians. Variations in clinical practice focus, exposure to diverse patient populations, and differing expectations for educational material likely influenced how relevance was evaluated. These inter-rater differences are expected in studies involving subjective assessments, as each clinician’s unique background and interpretive lens can shape how the relevance of educational content is perceived. A response that one provider considers highly relevant may not resonate the same way with another, particularly in a field as personalized and variable as female sexual health. Furthermore, the format of the Prosayla resources compared to the LLMs may have played an important role. Prosayla articles are written with the intention to cover broad educational topics for general consumption rather than be tailored to meet the needs of a specific patient. By contrast, LLMs can tailor responses to specific questions, enhancing perceived relevance. This adaptability may make AI-generated content appear more relevant to a patient’s specific inquiry compared to generalized written articles that may only partially touch on a patient’s unique concern. A potential use case for these LLMs would be for exploring their topics of concern before their visit with a clinician. The LLMs could serve as a medium of initial patient education so that patients can maximize their time with their clinician while confirming what they learned from the LLMs. Another potential use case could be after clinical visits, where patients use LLMs to clarify or reinforce key points discussed during the appointment, helping them better retain information and adhere to treatment recommendations.

The expanding use of AI in patient education carries both promise and challenges. AI offers advantages such as ease of access and adaptability to different reading levels.^9^ These attributes are particularly important in women’s sexual health, where stigma and discomfort can often create barriers to seeking information and care. However, privacy and confidentiality remain essential concerns when utilizing AI in sexual medicine.^10^ For example, AI-driven platforms such as chatbots should be designed to comply with data protection regulations like the General Data Protection Regulation (GDPR) in the European Union.^11^ The GDPR restricts automated decision-making and mandates explicit consent for processing health-related data. In addition, clinician oversight is necessary to make sure the information provided by AI tools remains accurate, relevant, and truly meets the needs of individual patients.^12^

Beyond these advantages, the use of AI in sexual health education also raises important ethical and safety considerations.^13^ While in this study, the double-blinded physician review regarding accuracy helped mitigate the possibility of “hallucinations” (incorrect statements) by the LLMs, it must be noted that LLMs can still produce outputs that are misleading or lack contextual nuance. Given this, it is critical that even high-performing models require clinical oversight to ensure complete factual integrity as well as cultural sensitivity. This critical safeguard, combined with clear data privacy protocols, is needed to ensure that the integration of AI into sexual health education remains safe and responsible.

A qualitative consideration of AI-generated responses underscores that, although major factual errors were not observed in our study, LLMs still run the risk of oversimplifying or omission of factors that carry clinical implications if interpreted without guidance. For example, Koh et al found that although ChatGPT was able to provide accurate advice about sexually transmitted infections, there were critical lapses in its ability to capture the nuanced clinical context that a physician would obtain during a patient encounter.^14^ This highlights the important point that clinician oversight remains essential to ensure that AI-generated information is interpreted accurately, placed within the proper clinical context, and translated into safe, individualized care.

One of the limitations of our study is that only two physicians were asked to evaluate responses. With only 2 raters and 12 questions, the study provides preliminary insights for an exploratory feasibility analysis rather than definitive statistical power. Larger datasets and additional evaluators will be necessary to strengthen future analyses. If more physicians and questions were utilized, the responses potentially could have been more accurate and precise. Furthermore, future studies could incorporate the patient perspective by including patient-generated ratings or usability assessments, which would provide valuable insight into the perceived usefulness of LLMs in the context of women’s sexual health. Questions in future studies should also include more colloquialisms to reflect the natural way people speak. In the future, using multiple different education websites to develop questions could also help minimize potential bias introduced by relying on a single comparator, such as Prosayla. Another potential limitation is that the Prosayla website’s primary use is to provide broad information rather than answer specific questions. The study also relied on Likert scale assessments, which are inherently subjective and may be influenced by each reviewer’s individual clinical experience and interpretation of relevance or accuracy. While this study compares the performance of multiple AI language models in patient education on women’s sexual health, it does not fully account for differences in training data and model update timelines. Even though the model of each LLM used in this study was the same for the duration of the steady and equivalent to each other in terms of development stage, results could have been more precise if all the queries were processed on the same day at the same time. Future studies should also incorporate multiple runs at different time points to better capture potential variability in outputs as models evolve. Finally, the study evaluated responses only in English, which limits the generalizability of the study to English speakers. Broader studies involving more diverse evaluators and patient populations could enhance future analyses.

## Conclusion

The findings from this study suggest that while traditional resources remain valuable, AI tools may offer advantages in delivering more personalized, contextually appropriate educational content for patients. This could be especially useful for patients intending to learn the basics of their condition before visiting a clinician or for reinforcement after a patient visits a clinician. However, mixed findings on the topic of relevance highlight that human oversight remains critical to ensure that patient concerns are resolved at the individual level, with the role of LLMs being supplemental rather than primary. Clinicians must continue to review AI-generated content carefully to ensure it provides reliable information and addresses the specific concerns of each patient. Continued research will be essential to better define the best practices for integrating AI and LLMs into patient education, especially in areas like female sexual health, where stigma, emotional sensitivity, and medical complexity often intersect.

Concluding with a call for structured integration strategies and clinical implementation frameworks, this work underscores the importance of establishing clear guidelines that responsibly and effectively incorporate AI into patient-facing educational tools. Future work should prioritize the development of secure AI platforms tailored to sexual health education, guided by clinicians and patient feedback, with safeguards for accuracy, cultural sensitivity, and data privacy.

## Declarations

**Ethics approval and consent to participate:** This study did not involve human participants, identifiable personal data, or clinical interventions, and therefore did not require ethical approval. All data analyzed were publicly available through Google Trends and published literature databases.

**Consent for publication:** Not applicable.

**Availability of data and materials:** The data generated and analyzed during this study are based on original evaluations conducted by two board-certified sexual medicine physicians, who independently rated AI-generated and Prosayla-sourced responses using a 6-point Likert scale to assess accuracy and relevance. Statistical analysis was performed using SPSS. All processed data tables and relevant materials are included within the published article. Additional information or clarification is available from the corresponding author upon reasonable request.
